# RETRACTION: Quercetin Improves Glucose and Lipid Metabolism of Diabetic Rats: Involvement of Akt Signaling and SIRT1

**DOI:** 10.1155/jdr/9878214

**Published:** 2026-04-28

**Authors:** Journal of Diabetes Research

RETRACTION: J. Peng, Q. Li, K. Li, L. Zhu, X. Lin, X. Lin, Q. Shen, G. Li, X. Xie, “Quercetin Improves Glucose and Lipid Metabolism of Diabetic Rats: Involvement of Akt Signaling and SIRT1,” *Journal of Diabetes Research*, 2017, 3417306, https://doi.org/10.1155/2017/3417306

The above article, published online on 12 December 2017 in Wiley Online Library (http://wileyonlinelibrary.com), has been retracted by agreement between the journal Associate Editor, Bright Starling Emerald; and John Wiley & Sons Ltd. The retraction has been agreed due to concerns identified with Figure 2, both directly and on PubPeer [1]. Specifically:

• In Figure 2a there are unexpected, overlapping regions between:
  o. The control panel and the Pioglitazone panel
  o. The control panel and the QU‐high panel
• In Figure 2c, the SIRT‐1 bands appear identical to the SIRT‐1 bands shown in Figure 2a of [2]
• In Figure 2c, the a‐Tubulin bands appear identical to the B‐actin bands shown in Figure 2a of [2]

After assessment of the concerns and the author’s response by the editorial board, the results and conclusions are not longer considered reliable, and the article is retracted.

The authors did not respond when informed of the retraction.
